# Greater audiovisual integration with executive functions networks following a visual rhythmic reading training in children with reading difficulties: An fMRI study

**DOI:** 10.1162/NETN.a.31

**Published:** 2025-10-30

**Authors:** Tzipi Horowitz-Kraus, Tasneem Ismaeel, Marwa Badarni, Rola Farah, Keri Rosch

**Affiliations:** Educational Neuroimaging Group, Faculty of Education in Science and Technology, Technion, Haifa, Israel; Faculty of Biomedical Engineering, Technion, Haifa, Israel; Kennedy Krieger Institute, Baltimore, MD, USA; Department of Psychology and Behavioral Sciences, Johns Hopkins University School of Medicine, Baltimore, MD, USA

**Keywords:** Audiovisual integration, Cognitive control, Rhythmic training, Dyslexia, Fluency

## Abstract

Reading difficulty (RD; dyslexia) is a developmental condition with neurological origins and persistent academic consequences. Children with RD often show deficits in audiovisual integration (AVI) and executive functions. Visual rhythmic reading training (RRT) has been associated with improvements in these domains, but it remains unclear whether such effects generalize to the resting-state brain activity. English-speaking children aged 8–12 years, including typical readers (TRs) and children with RD, were randomly assigned to an 8-week visual RRT or control math training group. Reading assessments and resting-state functional MRI data were collected before and after the intervention. Functional connectivity (FC) analyses examined AVI and its interaction with frontoparietal–cingulo-opercular (FP-CO) cognitive control networks during rest. Following RRT, children with RD showed significant improvements in reading fluency. The RRT group also demonstrated greater changes in AVI, which were associated with increased FC between FP-CO networks and sensory regions during the resting state. RRT improves reading performance and promotes enhanced integration between sensory and executive networks in children with RD, even in the absence of task demands. These findings support the role of RRT in fostering domain-general neuroplasticity beyond reading-specific contexts.

## Author Summary

Rhythmic reading training (RRT) improves reading performance and promotes enhanced integration between sensory and executive networks in children with reading difficulties, even in the absence of task demands. These findings support the role of RRT in fostering domain-general neuroplasticity beyond reading-specific contexts.

## INTRODUCTION

### Children With Reading Difficulties and Reading Fluency: The Involvement of Executive Functions

Reading difficulty (RD; or dyslexia) is a learning disorder affecting word recognition and spelling (IDA, 2011). While RD was traditionally related to deficits in phonological processing and slow and inaccurate reading (IDA, 2011), further studies revealed a multifaceted phenomenon. Children with RD often struggle with verbal visuospatial memory inhibition, switching, and processing speed (Barbosa et al., 2019; Horowitz-Kraus, 2014). These high-order cognitive abilities involve executive function (EF) (Anderson, 2002). Additionally, studies revealed a slower speed of processing in individuals with RD (Breznitz & Misra, 2003), thought to be a separate but related construct to EF important for the reading process (Anderson, 2002).

Several models have been proposed in an attempt to understand the reasons for RD by elucidating the basic components of reading development. One of these models is the Simple View of the Reading model (SVR) (Scarborough, 1998, 2001). The traditional SVR model suggests that reading development relies on three main components, including listening comprehension, word decoding, and reading comprehension (Scarborough, 1998, 2001). Further studies suggested that this model should also include reading fluency (i.e., the ability to read quickly and accurately; Breznitz, 2006) as well as EF (for review, see Horowitz-Kraus, 2023). Ongoing research in RD and reading fluency, or the speed of accurate reading, highlights the pivotal role of EF in this field (Kieffer & Christodoulou, 2020; Kim, 2020a, 2020b). These reading fluency deficits, that is, the challenges to read automatically and effortlessly, in individuals with RD were also found to be related to challenges in audiovisual integration (AVI) (Blau et al., 2010; Breznitz, 2006; Breznitz & Misra, 2003). The proposed mechanism was an inefficient integration between the auditory modality (i.e., the sounds of the letters) and the visual modality (i.e., the letters) (Breznitz & Misra, 2003). A recent neuroimaging study reported decreased functional connectivity (FC), defined as correlated brain activity, between the auditory and the visual networks during text reading in children with RD (Horowitz-Kraus et al., 2023). However, this study also revealed that encouragement to read faster increased the functional connections between the auditory and visual (Aud-Vis) networks and was related to greater reading comprehension (Horowitz-Kraus et al., 2023). One of the aspects affecting AVI is top-down EF skills, as well as the print characteristics (referred to as “bottom-up” aspects) (Zhou et al., 2020). These two aspects were found to be related to the time-binding window of the Aud-Vis modalities, which will then determine the efficiency of auditory-visual integration (Zhou et al., 2020). One of the suggested approaches to reduce the time binding window is by using rhythmic training, especially in the reading domain.

### Rhythmic Training, AVI, and Reading Fluency

Rhythmic reading training (RRT) uses time manipulation to enhance the processing of information arriving from the auditory modality, the visual, or both (Cancer et al., 2020, 2022). It was suggested that attention and EF are triggered and involved when rhythm is part of the training (Cancer & Antonietti, 2024). In the auditory domain, using a metronome during reading was considered an RRT (Cancer et al., 2020, 2022), suggesting to improve reading fluency skills in readers with RD. On the visual domain, visual RRT was referred to as a deleted text manipulation, which also involves speed components (e.g., the text is deleted in a speeded manner) while it monitors comprehension (Cecil et al., 2021; Horowitz-Kraus, 2015a; Horowitz-Kraus & Breznitz, 2014; Horowitz-Kraus, Cicchino, et al., 2014; Horowitz-Kraus, DiFrancesco, et al., 2015; Horowitz-Kraus & Holland, 2015; Horowitz-Kraus, Toro-Serey, & DiFrancesco, 2015; Horowitz-Kraus, Vannest, et al., 2014). Studies using this visual RRT suggested better word and contextual reading fluency and comprehension, with greater improvement observed in RD individuals than in typical readers (TRs) (Cecil et al., 2021; Horowitz-Kraus, 2015a; Horowitz-Kraus & Breznitz, 2014; Horowitz-Kraus, Cicchino, et al., 2014; Horowitz-Kraus & Holland, 2015; Horowitz-Kraus, Toro-Serey, & DiFrancesco, 2015; Horowitz-Kraus, Toro-Serey, & Holland, 2015; Horowitz-Kraus, Vannest, et al., 2014). Training with the visual RRT described above was also related to increased EF abilities as well as visual attention (Horowitz-Kraus & Holland, 2015).

Neurobiologically, English-speaking 8- to 12-year-old children with RD exhibited significantly greater activation and increased FC within visual processing regions (Horowitz-Kraus & Holland, 2015; Horowitz-Kraus, Vannest, et al., 2014; Taran et al., 2023), auditory-related regions and networks (Farah et al., 2024), and cognitive control regions and networks (e.g., cingulo-opercular [CO] network) (Horowitz-Kraus & Holland, 2015) following 8 weeks of visual RRT. Functional connections between the sensory and EF or attention networks were also found following training: auditory frontoparietal (FP) networks (Farah et al., 2024) (during a resting-state-like data) and visual processing–CO networks (Horowitz-Kraus & Holland, 2015; Taran et al., 2023). Despite the reported behavioral and neurobiological gains in cognitive and reading abilities following the visual RRT in children with RD, it is still unknown whether these gains are attributed to visual-auditory brain synchronization (Horowitz-Kraus, Toro-Serey, & DiFrancesco, 2015) and whether this synchronization is related to changes in the FC of EF networks. The goal of this study is to determine the changes in the FC of visual-auditory systems with changes in EF networks (CO-FP) following the visual RRT during a resting-state condition.

We hypothesize that the visual RRT will be related to increased between-network FC of the visual-auditory networks. We also suggest that this increase in the between-network FC will be moderated by increased between-network FC of cognitive control networks (CO-FP), indicative of improved integration and coordination between sensory and control systems. Additionally, we anticipate that children with RD will exhibit greater benefits from the intervention than TRs, both behaviorally and neurobiologically. To test these hypotheses, we will use a resting-state condition administered before and after the intervention.

## METHODS

### Participants

A total of 78 8- to 12-year-old English speakers participated in the current study, all healthy, right-handed, and neurologically intact (without psychiatric or developmental disorders, including attention-deficit/hyperactivity disorder). Children in the RD group demonstrated a score lower than 25% in at least two reading tests from a list of reading tests listed below (following Horowitz-Kraus et al., 2023; Taran et al., 2022, 2023). Written consent forms were provided for all children, and compensation was provided for their time and travel. The study was approved by the local institutional review board.

### Study Procedure

The participants were assigned to two reading groups: 41 TR and 37 children with RD. Participants were further randomized into two intervention types: (a) visual RRT (22 TRs including 16 males and six females; 21 children with RD including eight males and 13 females) and (b) a control training condition, that is, a computerized math training (19 TRs including eight males and 11 females; 16 children with RD including seven males and nine females). All children had at least average IQ scores (≥85) verified using the Test of Nonverbal Intelligence, 3rd Edition (TONI-III; Brown et al., 1997) and no history of a diagnosis of attention deficit hyperactivity disorder or currently elevated ADHD symptoms, which was confirmed using the Conners Questionnaire–III (Conners, 2008). See Table 1.

**Table 1.** Participant demographic and cognitive characteristics and baseline reading performance among children with RD and TR

Measure	RD (*n* = 37)	TR (*n* = 41)	Group comparison T(*p*)	95% confidence interval of the difference	
				Lower	Upper
***Participant characteristics***					
Sex (*n*, female: male)	15:22	17:24	—		
Age (years)	9.4(1.4)	10.1(1.41)	−1.97(0.052)	−1.295	0.005
Maternal education in years	17.5(2.92)	18.0(2.18)	−0.905(0.368)	−1.724	0.647
Nonverbal IQ (TONI-III, SS)	101.4(12.65)	106.3(9.0)	−1.949(0.055)	−9.983	0.111
Verbal abilities (PPVT, SS)	108.7(14.2)	122.3(13.7)	**−4.213(<0.001)**	**−20.011**	**−7.158**
Conners (parent report, T-score)	57.4(14.6)	50.5(13.4)	**2.14(0.035)**	**0.491**	**13.428**
***Reading measures***					
Contextual fluency (TOSREC index SS)	85.3(14.2)	104.6(18.2)	**−5.12(<0.001)**	**−26.930**	**−11.844**
Word reading fluency (TOWRE SWE SS)	82.4(12.5)	104.1(11.8)	**−7.69(<0.001)**	**−27.255**	**−16.037**
Non-word reading fluency (TOWRE PDE SS)	83.6(11.1)	104.6(9.7)	**−8.65(<0.001)**	**−25.784**	**−16.129**
Non-timed word reading (WJ-IV letter word SS)	90.2(14.7)	117.3(10.5)	**−9.13(<0.001)**	**−32.945**	**−21.146**
Non-timed non-word reading (WJ-IV word attack SS)	93.7(15.5)	119.3(15.5)	**−7.126(<0.001)**	**−32.742**	**−18.430**
Passage comprehension (WJ-IV SS)	85.9(10.5)	106.5(11.6)	**−8.01(<0.001)**	**−25.701**	**−15.461**

*Note*. Mean(*SD*) are reported unless otherwise noted. SS = standard score (*M* = 100, *SD* = 15); TOSREC = Test of Silent Reading Efficiency and Comprehension; TOWRE = Test of Word Reading Efficiency; SWE = Sight Word Efficiency; PDE = Phonemic Decoding Efficiency; TONI = Test of Nonverbal Intelligence; PPVT = Peabody Picture Vocabulary Test; WJ = Woodcock Johnson Tests of Achievement. Significant results (*p* < 0.05, corrected for multiple comparisons) are noted in **bold**.

The study employed a pre-/post-intervention design with RD and TR children randomized into two intervention groups: a visual RRT and a control training. Before the intervention, participants underwent baseline assessments, including cognitive assessments, behavioral and reading tests, and resting-state data collection. Following the baseline assessments, the participants completed visual RRT over 8 weeks, with three sessions per week lasting around 20 min per session. This time frame of 8 weeks was chosen in order to increase the magnitude of the effect of intervention (following Breznitz et al., 2013; Eden et al., 2004; Simos et al., 2007; Torgesen, 2005) and following previous experience using this training software (Horowitz-Kraus, 2015a, 2015b; Horowitz-Kraus, & Breznitz, 2014; Horowitz-Kraus, Cicchino, et al., 2014; Horowitz-Kraus & Holland, 2015; Horowitz-Kraus, Toro-Serey, & Holland, 2015; Horowitz-Kraus, Vannest, et al., 2014). The reading training (as well as the arithmetic control training) included different stimuli for each training session and, this way, avoid priming and practice effect. During the intervention period, participants’ progress was monitored through online monitoring in the software and the study team. After completing the intervention, participants underwent post-intervention assessments, including repeated cognitive assessments, reading tests (with different versions of tests when applied), and fMRI scans. All assessments and intervention sessions were conducted in a controlled environment to ensure consistency and minimize external influences.

### Behavioral Measures

To assess reading abilities, standardized English tests were administered to both children with RD and TRs. The Test of Word Reading Efficiency (TOWRE–Sight Word Efficiency; Torgesen et al., 1999) was used to evaluate orthographic processing, while the Comprehensive Test of Phonological Processing, 2nd Edition (CTOPP-II; Wagner et al., 1999) Elision subtest was used to assess phonological processing. Additionally, the Gray Oral Reading Test, 2nd Edition (GORT-II; Bryant et al., 2009) and the Test of Silent Reading Efficiency and Comprehension (TOSREC; Wagner et al., 2010) were used to measure reading accuracy (number of correctly read words), reading rate (speed), and comprehension. These assessments included both isolated word/non-word reading tasks to gauge orthographic and phonological processing and contextual reading tasks to evaluate reading accuracy and rate.

### Neuroimaging Data

Neuroimaging data were obtained using a Philips Ingenia 3 Tesla MRI scanner (Philips Healthcare, Best, Netherlands) at the Cincinnati Children’s Hospital Medical Center. A gradient echo-planar multi-band sequence was used for T2*-weighted fMRI scans with the following parameters: Time Repetition (TR)/Echo Time (TE) = 700/30 ms, matrix = 80 × 80, slice thickness = 3 mm, 48 slices. A high-resolution T1-weighted 3D anatomical scan was acquired using an Inversion Recovery-prepared turbo gradient-echo acquisition protocol with a spatial resolution of 1 × 1 × 1 mm^3^. The T1 scan parameters were a TR of 8.1 ms, TE of 3.7 ms, inversion time of 940 ms, and flip angle of 8°, with a field of view (FOV) of 22.4 × 25.6 × 16 cm, a 224 × 256 matrix, and a slice thickness of 1 mm.

Prior to the fMRI sessions, children were introduced to the MRI scanner environment and practiced lying still on the scanner bed to reduce movement (Horowitz-Kraus et al., 2023; Taran et al., 2022, 2023; Vannest et al., 2014). Foam pads were used around the head to help minimize motion during the scans. The stimuli were presented using an MRI-compatible audio-visual system (Avotec, SS3150/SS7100).

### Data Analyses

#### Behavioral data analysis.

Behavioral analysis was conducted using SPSS 27 (IBM, Armonk, New York, USA). Two-sample *t* tests examined baseline group differences in age, nonverbal and verbal abilities, and demographic measures.

To assess the intervention’s effect on reading measures in both groups, several 2 Group (children with RD vs. TRs) × 2 Training Type (RRT vs. control training) × 2 Time (pre-intervention; Test 1 vs. post-intervention; Test 2) repeated measures analyses of variance (RM-ANOVA) were conducted for the reading measures, corrected for multiple comparisons using the Bonferroni correction.

#### Neuroimaging data analysis.

##### Preprocessing.

The preparation of the fMRI data involved several steps using Statistical Parametric Mapping 12 (SPM12) and CONN functional connectivity toolbox, Version 22a (Whitfield-Gabrieli & Nieto-Castanon, 2012). The preprocessing process included realignment and unwarping of functional volumes; slice-timing correction; segmentation into gray matter, white matter, and cerebrospinal fluid (CSF); and normalization to the standard Montreal Neurological Institute space. An 8-mm Gaussian kernel (full-width at half-maximum) was used to spatially smooth the functional images, in alignment with established resting-state fMRI preprocessing standards (Freedman et al., 2020; Horowitz-Kraus, Toro-Serey, & DiFrancesco, 2015).

Head motion correction was performed using a rigid-body realignment algorithm in SPM12, which estimated six motion parameters for each volume (three translational and three rotational components; Friston et al., 1995). Potential motion-related artifacts were detected using the Artifact Detection Tools (ART) integrated in the CONN toolbox. Volumes exceeding a framewise displacement of 0.9 mm or a global signal deviation beyond 5 standard deviations were flagged as outliers. These identified volumes were subsequently modeled as nuisance regressors in the denoising step. ART also computed average framewise displacement and global signal change metrics across participants to quantify motion characteristics.

After spatial preprocessing, functional data were denoised using the anatomical CompCor (aCompCor) method (Behzadi et al., 2007). This process involved regressing out five principal components extracted from white matter and CSF time series, as well as the six motion parameters and their first-order derivatives. The cleaned BOLD signal was then subjected to temporal filtering using a band-pass range of 0.008 to 0.09 Hz, which isolates neural oscillatory activity while attenuating physiological and scanner-related noise (Baria et al., 2011).

**Figure 1.**
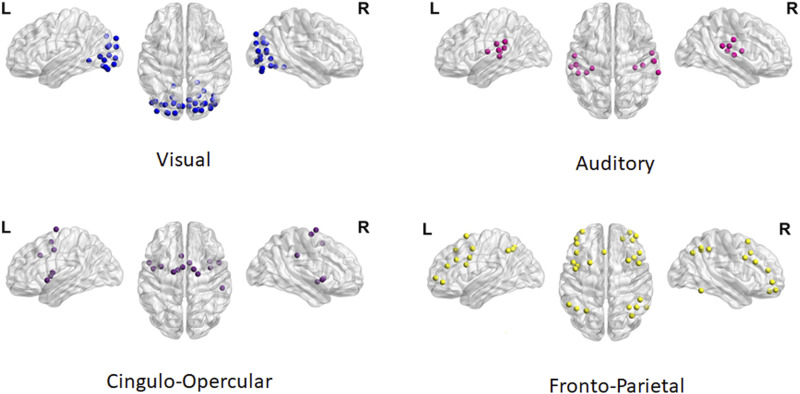
Spatial locations of the AV and EF networks included in the analyses.

Following spatial preprocessing, regions of interest (ROIs) related to reading (Aud-Vis) and EF (CO, FP) were defined using the Power Atlas (Power et al., 2011). Custom MATLAB-based programming was then incorporated to explore second-level FC within and between networks, calculated as the mean of the pairwise, Fisher-transformed, bivariate correlation coefficients for all ROIs within or between networks. FC within and between these networks was calculated for each group before and after the intervention. The selected networks are shown in Figure 1.

To determine the effect of the visual RRT on between-network FC, two 2 Group × 2 Training Type × 2 Time RM-ANOVAs were conducted for each of the FC values (CO-FP, Aud-Vis processing), as done for the behavioral measures.

### Pearson Correlation Analyses

To determine the relations between the differences in Aud-Vis FC and EF FC following training in both training and reading groups, a Pearson correlation was conducted. Finally, two additional Pearson correlations were conducted between the FC of the changes in Aud-Vis and in EF networks following training and reading gain across all children. Data were corrected for multiple comparisons using a Bonfferoni correction for all the analyses above.

## RESULTS

### Baseline Behavioral Differences Between RD and TR Groups

Independent *t* test analyses comparing reading measures at baseline between RD and TR groups revealed lower reading scores in children with RD. See Table 1.

### Effect of Interventions

Results of the 2 Group × 2 Training Type × 2 Time RM-ANOVA revealed a significant main effect of Group for all examined reading measures, including contextual reading fluency, word-level and non-word level reading fluency, non-timed reading tests, reading comprehension, and speed of processing, with higher scores for TRs relative to the RD group. A main effect of Time was also found for contextual fluency and comprehension, with higher scores following training versus prior to it. See Table 2 for the significant results.

**Table 2.** Results of the RM-ANOVA for reading behavioral measures from pre- to post-intervention in RD and TR groups

Skill	Measure	RD group				TR group				*F* tests
		RRT		Control (math)		RRT		Control (math)		
		Test 1	Test 2	Test 1	Test 2	Test 1	Test 2	Test 1	Test 2	
Contextual fluency	TOSREC (scaled score)	83.27 (3.97)	89.66 (6.6)	89.26 (4.35)	90.8 (4.24)	104.78 (3.51)	104.22 (3.42)	100.28 (3.68)	110.71 (3.58)	Time: *F*(1, 73) = 5.92, *p* = 0.017, *η*^2^ = 0.075
										Group × Training Type × Time: *F*(1, 73) = 4.7, *p* = 0.03, *η*^2^ = 0.06
Word reading fluency	TOWRE (SWE, scaled score)	84.36 (2.98)	85.47 (3.89)	80.75 (3.25)	84.93 (2.42)	101.26 (2.71)	101.47 (3.53)	103.52 (2.83)	99.71 (3.7)	**Group: *F*(1, 75) = 35.4, *p* < 0.001, *η*^2^ = 0.32**
Non-word reading fluency	TOWRE (PDE, scaled score)	83.94 (2.65)	83.31 (3.94)	84.25 (2.88)	83.06 (4.3)	102.78 (2.4)	102.43 (3.58)	103.76 (2.52)	104 (3.75)	**Group: *F*(1, 75) = 46.3, *p* < 0.001, *η*^2^ = 0.381**
Word-reading (untimed)	Letter word (WJ, standard score)	90.05 (3.29)	91.36 (3.02)	92.5 (3.59)	92.75 (3.3)	113.72 (3.06)	114.18 (2.81)	115.81 (3.13)	113.09 (2.88)	**Group: *F*(1, 74) = 57.0, *p* < 0.001, *η*^2^ = 0.435**
Non-word reading (no time constraints)	Word attack (WJ, standard score)	96.11 (3.73)	94.84 (3.4)	93.37 (4.0)	97.37 (3.7)	114.31 (3.46)	113.72 (3.16)	117.52 (3.55)	117.9 (3.23)	**Group: *F*(1, 74) = 40.0, *p* < 0.001, *η*^2^ = 0.351**
Reading comprehension	Reading comprehension (WJ, standard score)	85.15 (2.82)	87.57 (3.06)	88.56 (3.07)	91.68 (3.34)	105.72 (2.61)	108.18 (2.85)	103.25 (2.74)	106.85 (2.99)	**Group: *F*(1, 73) = 40.8, *p* < 0.001, *η*^2^ = 0.358**
										**Time: *F*(1, 73) = 8.8, *p* = 0.004, *η*^2^ = 0.158**
Speed of processing	Letter-naming (CTOPP, scaled scores)	7.11 (0.50)	7.32 (0.50)	6.19 (0.54)	7.5 (0.54)	8.59 (0.46)	9.00 (0.47)	8.66 (0.48)	8.62 (0.48)	Group × Training Type × Time: *F*(1, 74) = 3.5, *p* = 0.06, *η*^2^ = 0.045

*Note*. SS = standard score (*M* = 100, *SD* = 15); TOSREC = Test of Silent Reading Efficiency and Comprehension; TOWRE = Test of Word Reading Efficiency; SWE = Sight Word Efficiency; PDE = Phonemic Decoding Efficiency; TONI = Test of Nonverbal Intelligence; PPVT = Peabody Picture Vocabulary Test; WJ = Woodcock Johnson Tests of Achievement. Significant results (*p* < 0.05 without correction for multiple comparisons) are noted in **bold**.

### Neuroimaging Results

#### Baseline brain FC differences between RD and TR groups.

Independent *t* test analyses comparing baseline brain FC between the RD and TR groups showed no statistically significant differences, although the RD group exhibited numerically higher between-network connectivity. The comparison yielded a medium effect size for the CO-FP networks (*t* = 1.93, *p* = 0.057, *d* = 0.45) and a small effect size for the Aud-Vis networks (*t* = 1.38, *p* = 0.171, *d* = 0.31), suggesting potential group differences that warrant further investigation in larger samples.

### Effect of Intervention

**Figure 2.**
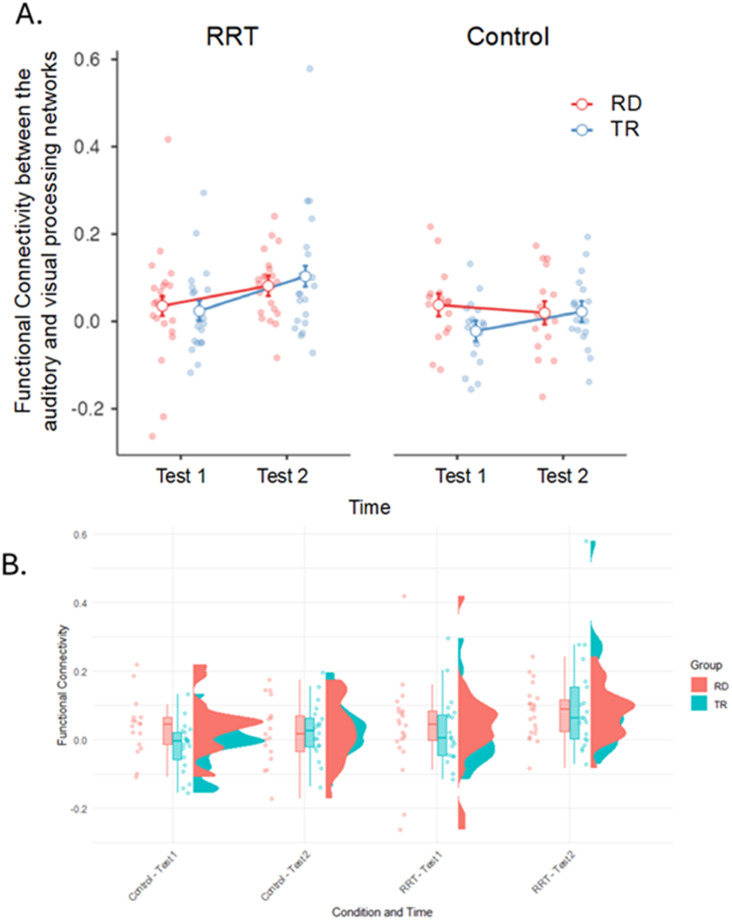
Differences in FC of the Aud-Vis networks before and after intervention in the RRT and control conditions. (A) Line plots show the mean FC values between auditory and visual networks at pre-intervention (Test 1) and post-intervention (Test 2), separated by intervention type (RRT vs. Control) and group (RD = children with reading difficulties; TR = typically reading children). Lines represent group-level trends; dots represent individual data points. (B) Raincloud plot illustrating the distribution of individual FC values by condition and timepoint. Violin plots display kernel density estimates, boxplots show medians and interquartile ranges, and raw data points are jittered. Greater post-intervention connectivity is observed in the RRT condition, particularly among children with reading difficulties.

**Figure 3.**
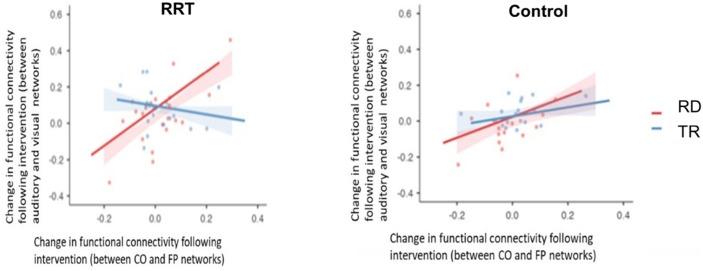
An interaction between the group and training type for the change in FC between the auditory-visual networks and EF networks.

Results of the 2 (Group) × 2 (Training Type) × 2 (Time) RM-ANOVA revealed two nonsignificant but potentially meaningful two-way interactions for Aud-Vis FC. A Group × Time interaction approached significance, *F*(1, 72) = 2.8, *p* = 0.097, *η*^2^ = 0.038 (Figure 3), with post hoc *t* tests indicating greater improvement in Aud-Vis FC among children with RD (*p* = 0.005, *d* = 0.75) compared with typically reading peers (*p* = 0.089, *d* = 0.44). Additionally, a Training Type × Time interaction showed a similar pattern, *F*(1, 72) = 3.1, *p* = 0.084, *η*^2^ = 0.040, suggesting greater gains in Aud-Vis FC for children who completed the RRT compared with those in the control (math) training. A significant main effect of Training Type was also observed for the CO-FP networks, with a greater increase in between-network FC following the RRT. See Table 3 and Figure 2 for the between-network FC values.

**Table 3.** Results of the RM-ANOVA for between FC of neuroimaging networks from pre- to post-intervention in RD and TR groups

Network	RD				TR				*F*
	RRT		Control		RRT		Control		
	Test 1	Test 2	Test 1	Test 2	Test 1	Test 2	Test 1	Test 2	
CO-FP	0.086 (0.073)	0.102 (0.094)	0.084 (0.081)	0.062 (0.091)	0.071 (0.122)	0.088 (0.112)	0.018 (0.082)	0.038 (0.052)	Training Type: *F*(1, 72) = 4.1, *p* = 0.048, *η*^2^ = 0.053
Aud-Vis	0.035 (0.136)	0.082 (0.078)	0.038 (0.087)	0.020 (0.098)	0.024 (0.101)	0.103 (0.150)	−0.022 (0.074)	0.022 (0.081)	Time × Group: *F*(1, 72) = 2.836, *p* = 0.097, *η*^2^ = 0.038
									Time × Training Type: *F*(1, 72) = 3.075, *p* = 0.084, *η*^2^ = 0.041

### Correlation Between Differences in Auditory-Visual Processing FC and Cognitive Control FC Following Training in Both Training and Reading Groups

A significant correlation between the change in resting-state FC between the auditory and visual networks and the change in FC between the CO and FP networks following training with the RRT, *r*(21) = 0.643, *p* = 0.002, versus a control condition, control: *r*(16) = 0.453, *p* = 0.08, in children with RD. These relationships were not observed in TR children, RRT: *r*(20) = −0.191, *p* = 0.42; control: *r*(19) = 0.329, *p* = 0.17. See Figure 3.

### Correlations Between the Changes in FC Between the Auditory-Visual Processing Networks and in the EF Networks and Reading Gain Across All Children

Greater change in FC between the EF networks (CO-FP) following training was positively correlated with changes in word reading fluency (TOWRE, SWE: *r* = 0.205, *p* = 0.038). Moreover, greater change in FC between the auditory and visual processing networks was correlated with changes in word reading fluency (TOWRE, SWE: *r* = 0.205, *p* = 0.038). Overall, a greater increase in FC between the auditory-visual processing networks and between EF networks was related to greater improvement in fluent word reading across all participants. Following correction for multiple comparisons, only *p* values lower than 0.025 survived the correction.

## DISCUSSION

The goal of this study was to determine the effect of training with a visual RRT (vs. a control math training) on AVI and functional connections with the cognitive control networks in children with RD versus TRs.

We hypothesized that following intervention, greater gain in reading abilities would be observed in the reading training group and in the RD versus TR group. We also hypothesized that children who received the RRT would show greater AVI (i.e., greater audio-visual FC) and greater synchronization between the sensory and EF/attention system (i.e., increased FC between the corresponding networks) and that this effect would be larger in the RD versus TR group. Finally, we hypothesized that changes in the functional connections between the sensory and EF networks would be correlated with greater reading improvements.

### The Role of AVI and Cognitive Control in Supporting Improved Reading Fluency

The SVR model emphasizes the importance of listening comprehension and word decoding for reading comprehension (Gough & Tunmer, 1986), whereas word decoding relies on Aud-Vis processing (Dehaene, 2009). Although EF and reading fluency do not have a specific role in this model (Horowitz-Kraus, 2023), more recent studies support the role of both EF (Kim, 2020b) and reading fluency (Kieffer & Christodoulou, 2020) as part of the SVR model. It was further suggested that not only are the Aud-Vis modalities critical, but the quick and efficient integration of information processed in these modalities is also essential for reading fluency (Breznitz, 2006; Breznitz & Misra, 2003). EF was found to facilitate this AVI process (Zhou et al., 2020), with the possibility that different modalities of RRT (e.g., auditory vs. visual) may be similarly effective as this type of training engages both cognitive control abilities (with increased speed of processing and attention orienting) and AVI (Horowitz-Kraus et al., 2023). The results of the visual RRT in the current study support this mechanism, as greater FC between the Aud-Vis networks and cognitive control networks was found in the RD group, which was related to improved reading fluency. These results support the importance of AVI and the engagement of EF networks in improving reading fluency in children with RD. The visual RRT manipulation involves accelerating the deletion pace to increase the engagement and integration between the Aud-Vis networks and the EF networks, thereby allowing faster processing of words in a context (i.e., sentence). Whether their reading strategy changes from letter decoding to an orthographical one is still a matter of debate, which can be addressed using eye-tracking studies in further research. Whether this AVI is specific to reading materials or generalized to nonlinguistic information is also yet to be known and warrants additional studies.

### Dyslexia—Is It Only an AVI Issue?

Several theories were raised to point out the causes of RD or dyslexia, with one theory that may explain most challenges: a deficit in AVI in these individuals (Breznitz, 2006; Breznitz & Misra, 2003; Zhou et al., 2020). The ability to quickly and accurately integrate information from the Aud-Vis modalities is critical for most cognitive tasks. Hence, it is not surprising that several neurodevelopmental groups, such as those with epilepsy, autism spectrum disorder, and attention-deficit/hyperactivity disorder, show difficulties in AVI (Zhou et al., 2020). This challenge in AVI in children with RD can explain their orthographical difficulty (Schlaggar & McCandliss, 2007; Share, 1995), inability to efficiently match the sound to letters (Snowling, 1995), and slow motor learning (Decarli et al., 2024). Combined with their lower EF abilities (Horowitz-Kraus, 2014), it might be that disruptions in AVI and weaknesses in EF may contribute to the severity of the other challenges observed in children with dyslexia. Encouragingly, rhythmic training seems to manipulate both components (AVI and EF), which is related to improved reading fluency skills. The effect on AVI was found in both RD and TR groups. However, the combined effect on AVI and EF together was specific to the RD group. It might be that due to the challenge children with RD share in EF, they need to engage those networks as well in order to read more fluently. Whether the effect is more pronounced when a visual RRT is used versus an auditory one is still unknown. Moreover, as in those with RD, EF is also engaged following training; whether an additional EF warm-up before the actual visual RRT is administered will increase the gain from training with respect to ready fluency is also unknown and warrants additional research. Finally, understanding how interindividual variability in AVI and EF relates to reading performance is an important next question for guiding personalized intervention approaches.

### Study’s Limitation

This study has several limitations: First, although we included a control group of children trained in math, the number of children in this group was lower than in the actual training group. In addition, math also has an EF component, which may have reduced the differential effect of the intervention in the RRT relative to the math control group. Hence, the ideal control training to directly test the effect of the RRT would be a control condition in which children read the same materials but without rhythmic manipulation (i.e., text deletion). Finally, the relatively small sample size within each intervention group may reduce statistical power and increase the likelihood of false negatives. This is particularly relevant given the individual variability often seen in children with reading difficulties. To address this, we report 95% confidence intervals alongside all key statistics to provide a clearer picture of effect size precision. Future studies with larger samples are needed to confirm and extend these findings.

### Conclusions

The results of the current study demonstrate the role of the integration between the Aud-Vis networks and EF in reading fluency and improvement in reading fluency with a visual RRT. These findings support theories highlighting the importance of EF in AVI (Zhou et al., 2020) and the role AVI in reading fluency (Breznitz, 2006). Collectively, these findings can serve as the basis for developing different interventions targeting AVI or EF to improve reading fluency in populations with RD.

## AUTHOR CONTRIBUTIONS

Tzipi Horowitz-Kraus: Conceptualization; Data curation; Formal analysis; Funding acquisition; Investigation; Methodology; Resources; Software; Supervision; Validation; Visualization; Writing – original draft; Writing – review & editing. Tasneem Ismaeel: Conceptualization; Funding acquisition; Methodology; Writing – original draft. Marwa Badarni: Formal analysis; Methodology; Writing – original draft. Rola Farah: Data curation; Formal analysis; Investigation; Methodology; Supervision; Writing – original draft; Writing – review & editing. Keri Rosch: Conceptualization; Data curation; Formal analysis; Investigation; Methodology; Software; Visualization; Writing – original draft; Writing – review & editing.

## FUNDING INFORMATION

Tzipi Horowitz-Kraus, National Institute of Child Health and Human Development (https://dx.doi.org/10.13039/100000071).

## DATA AVAILABILITY STATEMENT

Data will be available upon request from the corresponding author (Horowitz-Kraus).

## TECHNICAL TERMS

Executive functions (EFs): A collective term referring to higher order cognitive processes that regulate reasoning, problem-solving, and goal-directed planning through top-down control mechanisms.
Cingulo-Opercular Network (CO): A neural network encompassing the dorsal anterior cingulate cortex, anterior insula, and dorsolateral prefrontal cortex, associated with sustained cognitive control and the regulation of goal-directed behavior.
Fronto-Parietal Network (FP): A brain network responsible for initiating task-related cognitive sets and coordinating large-scale brain activity, supporting functions such as complex reasoning, long-term memory, and reading.
